# Social and emotional characteristics of girls and young women with DDX3X-associated intellectual disability: a descriptive and comparative study

**DOI:** 10.1007/s10803-022-05527-w

**Published:** 2022-05-10

**Authors:** Elise Ng-Cordell, Anna Kolesnik-Taylor, Sinéad O’Brien, Duncan Astle, Gaia Scerif, Kate Baker

**Affiliations:** 1 Department of Psychology, University of British Columbia, Vancouver, Canada; 2 MRC Cognition and Brain Sciences Unit, University of Cambridge, Cambridge, United Kingdom; 3 Department of Experimental Psychology, University of Oxford, Oxford, United Kingdom; 4 Department of Medical Genetics, University of Cambridge, Cambridge, United Kingdom

**Keywords:** DDX3X, Intellectual disability, Autism, Anxiety, Self-injurious behaviour

## Abstract

*DDX3X* variants are a common cause of intellectual disability (ID) in females, and have been associated with autism spectrum disorder and emotional-behavioural difficulties. In this study, we compared phenotypic data for 23 females with *DDX3X* variants, to 23 females with ID and other genetic diagnoses. We found a wide range of adaptive, social and emotional function within the *DDX3X* group. Autism characteristics did not differ between *DDX3X* and comparison groups, while levels of anxiety and self-injurious behaviour (SIB) were significantly higher in the *DDX3X* group. Within the *DDX3X* group, adaptive function, autism characteristics, anxiety and SIB scores were positively correlated, with evidence for group-specific associations with SIB. Future work is warranted to explore the multilevel mechanisms contributing to social and emotional development in individuals with *DDX3X* variants.

**Supplementary Information:**

The online version contains supplementary material available at 10.1007/s10803-022-05527-w.

## Background

At least 60% of individuals with severe intellectual disability (ID) have an underlying genetic diagnosis (Gilissen et al., 2014). With advances in genome-wide diagnostic technologies, it is increasingly possible to diagnose genetic causes of ID, representing an opportunity to study causal factors influencing development and lifespan experiences among individuals with ID. With ever increasing numbers of rare and ultra-rare genetic diagnoses associated with ID, it is especially important to place the cognitive and behavioural characteristics associated with each diagnosis in the context of expectations for ID in general, and to identify factors associated with variability within genetic diagnosis groups. Comparative studies of behavioural characteristics can thus highlight whether features occur at elevated rates, or whether they occur in line with expectation for ID more generally (O’Brien et al., 2019). In addition, studying relationships between adaptive, social, and emotional dimensions can be a starting point towards understanding mechanisms contributing to mental health (e.g., mood regulation, anxieties, behaviours that challenge) across syndromes.

*DDX3X* loss-of-function variants are one of the more common single-gene causes of ID, affecting an estimated 1–3% of females with unexplained ID (Snijders-Blok et al., 2015; Wang et al., 2018) and more rarely affecting males (Nicola et al., 2019). As availability of genetic testing increases, numbers of individuals diagnosed with *DDX3X* variants are likely to rise considerably. The *DDX3X* gene is located at chromosome Xp11.4, and encodes an ATP-dependent DEAD-box RNA helicase (Park et al., 1998; Kim et al., 2001) involved in multiple aspects of RNA metabolism including splicing and transport (Abdelhaleem, 2005), mitochondrial DNA production (Wang et al., 2018), and the Wnt/Beta-catenin signalling pathway (Snijders-Blok et al., 2015). Animal models have shown that *DDX3X* is highly expressed in radial glial progenitors and neurons, and promotes cortical development by regulating neurogenesis and neuronal migration (Chen et al., 2016; Lennox et al., 2020). Neuroimaging has highlighted diverse neuroradiological features (e.g., corpus callosum abnormalities, ventricular enlargement, cortical dysplasia, polymicrogyria), reflecting the regulatory functions of *DDX3X* during multiple phases of brain development (Lennox et al., 2020; Scala et al., 2019; Kellaris et al., 2018). Hence, there are diverse molecular, cellular and neural systems mechanisms by which pathogenic *DDX3X* variants may influence emergent cognition and behaviour.

Previous studies of individuals with *DDX3X* variants have primarily relied upon retrospective collation of clinical records, and have described a set of variable and complex clinical characteristics. Common physical features include cardiac and respiratory problems (e.g., sleep apnoea, congenital heart disease) and musculoskeletal disorders (e.g., joint hypermobility, scoliosis). A range of neurodevelopmental characteristics have been reported, encompassing global cognitive impairments from mild to severe ID, autism, hyperactivity, aggression, and motor stereotypies (Snijders-Blok et al., 2015; Wang et al., 2018; Lennox et al., 2020; Scala et al., 2019; Kellaris et al., 2018; Dikow et al., 2017; Beal et al., 2019).

Detailing the social and emotional characteristics of individuals with *DDX3X* variants, including factors underlying variation in outcomes, is important for understanding the support needs of diagnosed individuals as well as their families and caregivers (Stewart et al., 2017; Baker et al., 2021). Lennox et al. (2020) reported that among 53 individuals with *DDX3X* variants, average adaptive ability fell within the moderate ID range, while autism characteristics assessed via the Social Responsiveness Scale (Constantino & Gruber, 2012) and the Social Communication Questionnaire (Rutter et al., 2003) were moderately elevated above general population norms (not stratified for ID). Further, they reported that missense variants (changes to a single amino acid within the DDX3X protein) were associated with more severe clinical outcomes than protein truncating variants (changes to the length of the DDX3X protein, likely to result in reduced protein availability) across neuroanatomical, neurological, adaptive and behavioural domains. Elsewhere, Tang et al. (2021) characterized behavioural profiles of 15 individuals (14 females) with *DDX3X* variants via systematic post-diagnostic assessments of intellectual and adaptive functioning, sensory processing, autism and behavioural comorbidities. Based on diagnostic clinical assessments, 80% of individuals with *DDX3X* variants met diagnostic criteria for ID, 60% for autism spectrum disorder, and 53% for attention deficit / hyperactivity disorder (ADHD). Comparing scores on standardized questionnaire measures to population norms revealed high rates of sensory processing abnormalities such as sensory-seeking behaviours and sensory hypo-reactivity. In addition, 14% of individuals reported elevated anxiety above a likely clinically-relevant level. Comparing behavioural profiles across variant types revealed that missense variants and in-frame deletions were associated with poorer language, motor and adaptive outcomes compared to protein-truncating variants (Tang et al., 2021). Further, a smaller proportion of individuals with protein-truncating variants met diagnostic criteria for autism, although this difference was not significant and may reflect expected differences between groups with different adaptive abilities.

In summary, research to date points towards prevalent and diverse social and emotional difficulties in individuals with *DDX3X* variants. Moreover, variant type appears to be one aetiological factor underlying variability in adaptive, social and emotional characteristics. However, the distinctiveness of these characteristics from other causes of ID in females with a similar range of global adaptive severity, and relationships between adaptive-social-emotional dimensions, have not been established. Therefore, in the current study, we aimed to (1) describe the clinical, social, and emotional characteristics of girls and women with *DDX3X* variants, (2) identify social and emotional features that differentiate girls and women with *DDX3X* variants from those with other monogenic causes of ID, and (3) explore predictors (variant type, ID severity, autism characteristics) of social and emotional outcomes among girls and women with *DDX3X* variants.

## Methods

### Participants and recruitment

Individuals with a pathogenic or likely pathogenic variant in *DDX3X* were identified via clinical genetics services across the UK, the IMAGINE-ID (Intellectual Disability & Mental Health: Assessing the Genomic Impact on Neurodevelopment) Study (https://imagine-id.org/), or the *DDX3X* Support UK Group (https://ddx3xsupportuk.co.uk/). *DDX3X* variant interpretation had been carried out by clinical laboratories for all participants prior to entry to this study. Clinicians were given recruitment packs to distribute to family members of each potential participant. Members of the IMAGINE-ID Study and the *DDX3X* Support UK Group were provided with information and contact details for the study. The families of 23 girls and women volunteered to take part (n = 8 via clinical genetics services, n = 6 via the IMAGINE-ID Study, and n = 9 via the *DDX3X* Support UK Group). All 23 participants had been diagnosed with *DDX3X* variants through research or clinical pathways via whole exome sequencing or gene panel testing. Of the 20 participants for whom we had variant details, 7 had missense variants and 13 had protein truncating variants (protein truncating variants included frameshift, nonsense and splice site variants; Supplementary Information 2). The same procedures were followed to recruit a comparison group of 23 girls and women with ID of monogenic origin (ID-comparison group), represented by pathogenic variants in 11 different genes (Supplementary Information 1).

### Measures and Procedure

Assessments were conducted via parent questionnaires and interviews, which were carried out at participants’ homes for 12 families, and over the telephone or video conference for the remaining 11 families. Respondents were either mothers (n = 14; 61%), fathers (n = 2; 9%) or mothers and fathers together (n = 7; 30%).

#### Medical history

A study-specific Medical History Interview (MHI) gathered information about each individual’s perinatal history, infancy and childhood health, neurological symptoms and developmental milestones.

#### Adaptive functioning

The Vineland Adaptive Behaviour Scales (VABS) is a standardised assessment of adaptive functioning commonly used to evaluate neurodevelopmental disorders and ID. Scores can be obtained for adaptive functioning level on four domains: Communication, Daily Living Skills, Socialization, and Motor. Domain scores are summed to create a global Composite score (not including motor function). Raw scores are converted to standard scores, which are used to categorise level of impairment as borderline, mild, moderate, severe, or profound. Participants completed either the second edition, interview form (Sparrow et al., 2005; *DDX3X* n = 10; ID-comparison n = 23), or the third edition, parent/caregiver form (Sparrow et al., 2016; *DDX3X* n = 13); this was due to a second wave of recruitment occurring after publication of the VABS third edition).

#### Autism characteristics

The Social Responsiveness Scale, second edition (SRS; Constantino & Gruber 2012) is a parent-report questionnaire assessing social behaviours that are characteristic of autism. There are five subscales (Social Awareness, Social Cognition, Social Communication, Social Motivation, and Restricted Interests and Repetitive Behaviour), which are summed to create a total score. Raw scores are converted to T-scores, stratified by gender, and can be categorised as mildly, moderately, or highly elevated relative to population norms.

The Autism Diagnostic Interview – Revised (ADI-R; Lord et al., 1994) is a clinical interview assessing current and early (age 5) symptoms of autism. It is widely used to support autism evaluations in clinical and research settings (Lebersfeld et al., 2021). It comprises four domains (Language and Communication, Social Development and Play, Interests and Behaviours, and General Behaviours). The ADI-R was administered to seven participants in the *DDX3X* group; all families of DDX3X participants were invited to participate in this interview, but a shortened data collection timeline due to the COVID-19 pandemic meant that it was not feasible to conduct the ADI-R with all participants.

#### Emotional and behavioural difficulties

The Developmental Behaviour Checklist – second edition (DBC; Gray et al., 2018) is a parent-report questionnaire that assesses emotional and behavioural difficulties. Unlike other commonly used rating scales, the DBC was designed specifically to assess difficulties in individuals with ID. It includes five subscales assessing difficulties across five areas (Disruptive, Self-Absorbed, Communication Disturbance, Anxiety and Social Relating). Scores on the five subscales can be summed to calculate a Total Behavioural Problems score. Raw scores are converted to T-scores, stratified by ID severity (which was determined by participants’ VABS Composite scores). T-scores are used to indicate level of concern (Little, Moderate, Serious). An additional scale for self-injurious behaviours (SIBs) was calculated by summing raw scores of three DBC self-injury items (head banging; self-hitting/biting; skin picking/scratching).

### Analysis Plan

Raw subscale/domain and total scores on the VABS, SRS, and DBC were converted to standardised scores based on published normative data, and distributions of all scores examined for normality using Kolmogorov-Smirnov and Shapiro-Wilk tests prior to parametric or non-parametric analyses as appropriate.

#### Aim 1

To describe the clinical, adaptive, social, and emotional characteristics of participants with *DDX3X* variants, reports from the MHI, VABS, SRS, and DBC were summarised.

#### Aim 2

To identify adaptive, social, or emotional characteristics that might differentiate individuals with *DDX3X* variants from individuals with other causes of ID in females, the *DDX3X* group’s scores on the VABS, SRS and DBC were compared to those of the ID-comparison group. Univariate analyses (F-tests) were conducted to compare scores on the VABS, as this allowed the VABS form (i.e., second or third edition) to be controlled for. As scores from several subscales of the SRS (Social Awareness) and DBC (Anxiety) were not normally distributed, non-parametric (Mann-Whitney U / Wilcoxon rank-sum) tests were used to compare scores on the SRS and DBC.

#### Aim 3

To explore relationships between age, ID severity (VABS Composite), autism characteristics (SRS Total), anxiety (DBC Anxiety), and SIBs, pairwise correlations were conducted within the *DDX3X* and ID-comparison groups separately. To identify potentially distinct within-group relationships, the strength of correlations were compared via Fisher’s z transformation, and interactions examined in follow-up exploratory linear regression analyses. To examine whether variant type (missense vs. protein truncating) predicted levels of autism characteristics, anxiety and SIBs within the *DDX3X* group, a series of general linear models, with variant type as a fixed factor, were conducted.

## Results

### Aim 1: Describing the *DDX3X* group

*Clinical and developmental characteristics.* Clinical and developmental characteristics, taken from the MHI, are summarized in Table 1. Impairments affecting nearly all participants (> 90%) included delays in achieving communication and motor milestones, as well as gastrointestinal problems (most commonly feeding difficulties in infancy, and ongoing, chronic constipation). Common impairments (affecting > 50% of participants) included complications during pregnancy and childbirth, hypotonia during infancy, hearing and vision impairments, sleeping difficulties, movement disorders, musculoskeletal abnormalities, and self-injurious behaviours. Characteristics that were observed in < 50% of participants included sensory hypo/hyper-sensitivities (including a high pain threshold or sensory-seeking behaviours), respiratory and cardiological issues, skin conditions, minor congenital abnormalities, and epilepsy.

**Table 1:** Clinical phenotypes summary obtained from Medical History Interview (MHI)

Clinical feature / MHI item	Frequency of feature (n/23)	Frequency of feature (%)	Subtypes and frequencies (n)
**Gastrointestinal**	22	96	*Infancy*: Latching / sucking difficulties (14), Reflux (9), Choking (3) *Current / childhood*: Constipation / Immotile gut (8), Overeating (3), Overfills mouth (2), Incontinence (1) Cyclical vomiting (1)
**Delayed communication milestones**	21	91	Mild = using words and phrases (11), Moderate = using single words only (5), Severe = not using any words (4), Unable to classify as under age 5 years or insufficient information (1)
**Delayed motor milestones**	21	91	Mild = walked by 3 years (15), Moderate = walked by 5 years (6)
**Prenatal, Perinatal, and Neonatal complications**	15	65	*Prenatal*: Ultrasound abnormalities (5), Small foetus (3), Preeclampsia (1) *Perinatal*: Foetal heart rate fluctuation during delivery (4), Breech positioning (3), Umbilical cord abnormalities (3), *Neonatal*: Excessive crying (4), Jaundice (1), Meningitis (1)
**Hearing / Auditory**	14	61	Recurrent ear infections in infancy / early childhood, often leading to partial hearing loss (8), Hypersensitivity to sounds (8)
**Ophthalmic**	13	57	Far-sightedness / hyperopia (3), Cortical visual impairment (3), Squint (2), Nystagmus (1), Astigmatism (1), Abnormal pupil dilation (1), Poor depth perception (1), Blocked tear ducts (1), Eye tracking difficulties (1)
**Infantile hypotonia**	13	57	Parents usually noticed poor head control and ability to sit up during first year
**Movement problems**	13	57	Poor balance and coordination (10), Stiff gait (7), Stereotypies (3), Tremor (2), Ataxia (1), Dystonia (1)
**Sleep difficulties**	13	57	*Infancy*: Hypersomnia (4), Hyposomnia (2) *Current / childhood*: difficulties falling and staying asleep, early waking (9)
**Self-injury**	13	57	Hitting / biting (13), Skin scratching / picking (13), Head banging (11), Hair pulling (2)
**Musculoskeletal**	13	57	Joint Hypermobility (7), Hypotonia in limbs (4), Scoliosis (4), Inward-turning feet (2), Bone deficiency (1)
**Sensory**	10	43	Parents often reported a combination of sensory-seeking tendencies and sensory aversions, as well as a high pain tolerance.
**Respiratory / cardiology**	10	43	Frequent colds / respiratory infections (5), Heart murmur (4), Hay fever (3), Unspecified heart problems (2), Narrow heart valve (1), Asthma (1), Sleep apnoea (1)
**Skin conditions**	8	35	Eczema (6), Impetigo (1), Palmoplantar keratosis (1)
**Minor congenital abnormalities**	5	22	Inguinal hernia (2), Finger abnormalities (2), Adrenal hyperplasia (1), Cleft palate (1)
**Epilepsy**	6 diagnosed 8 suspected	26 35	*Diagnosed*: Absence seizures (2), Generalized seizures (1), Infantile spasms (1), Reflux anoxic seizures (1), Unspecified (3) *Suspected*: Absences / isolated seizures in past (8)
**Other**	7	30	Allergic reactions/oedema (3), Recurrent UTIs (2), Kidney Stones (1), Diabetes (1), Poor circulation in extremities (1), stereotypic laughter (1)

*Adaptive functioning.* Adaptive functions and social and emotional characteristics are summarized and displayed in Table 2. Global adaptive ability was measured using the VABS Composite score. Mean composite score was 53.09 (SD = 14.42; moderate ID range), although level of impairment ranged from borderline to profound (borderline n = 2, mild n = 9, moderate n = 8, severe n = 3, profound n = 1). Following published guidelines (Sparrow et al., 2005, 2016), we examined within-individual differences in domain-level scores (in Communication, Daily Living Skills, and Socialisation domains) to identify areas of relative strength or weakness. Communication (receptive, expressive, and writing skills) was a relative weakness for seven out of twenty-three participants (and a relative strength for one). Daily Living Skills (personal, domestic, and community skills) were a relative weakness for seven out of twenty-three participants (and a relative strength for one), while Socialisation (interpersonal relations, play and leisure, and coping skills) was an area of relative strength for eight out of twenty-three participants (Socialisation was not an area of weakness for any of the 23 participants).

**Table 2:** Adaptive, social and emotional characteristics

	*DDX3X*			ID-comparison			Group difference
	**N**	**Mean (Std Err)**	**Range**	**N**	**Mean (Std Err)**	**Range**	**DDX3X - ID-Comparison**
**Age**	23			23			
		12.87 (5.50)	3.07–22.43		13.20 (5.15)	6.72–26.28	t(44) = − 0.21, p = .84, d = 0.06, 95% C.I [-3.50, 2.84]
**VABS**	23			23			
ABC		53.09 (14.42)	20–74		55.61 (11.78)	34–79	F = 0.12, p = .74, η^2^ = 0.00, 95% C.I [-11.83, 8.46]
Communication		50.26 (17.41)	20–79		57.65 (14.25)	33–89	F = 0.00, p = .97, η^2^ = 0.00, 95% C.I [-12.04, 11.53]
Daily Living		48.65 (16.25)	20–73		54.74 (14.99)	30–83	F = 1.02, p = .32, η^2^ = 0.02, 95% C.I [-18.12, 6.04]
Socialization		57.48 (15.17)	20–78		59.22 (11.03)	42–88	F = 0.01, p = .51, η^2^ = 0.00, 95% C.I [-9.81, 10.58]
Motor	19	67.79 (11.87)	45–97		66.26 (17.75)	28–121	F = 0.19, p = .67, η^2^ = 0.01, 95% C.I [-9.39, 14.46]
**DBC2**	21			23			
Total		58.86 (16.03)	36–106		53.57 (12.11)	34–83	W = 283, p = .34, r = .15, 95% C.I [-0.4.00, 12.00]
Disruptive		54.14 (16.11)	38–112		50.22 (11.33)	35–77	W = 276, p = .42, r = .12, 95% C.I [-5.00, 9.00]
Self-absorbed		63.90 (17.66)	39–104		56.22 (12.96)	37–83	W = 298.50, p = .18, r = .20, 95% C.I [-3.00, 17.00]
Communication		59.29 (19.46)	42–132		55.78 (10.84)	36–81	W = 255, p = .76, r = .05, 95% C.I [-0.7.00, 8.00]
Anxiety		55.57 (10.57)	38–83		50.04 (12.25)	36–77	W = 335.5, p = .03, r = .33, 95% C.I [1.00, 13.00]
Social relations		51.67 (11.70)	36–76		51.04 (10.31)	38–73	W = 241.50, p = 1.00, r = .00, 95% C.I [-7.00, 8.00]
SIBs ^a^		2.67 (0.50)	0–6		1.22 (0.32)	0–6	W = 326.50, p = .04, r = .31, 95% C.I [0.00, 3.00]
**SRS**	21			22			
Total		77.38 (14.08)	46–98		76.82 (14.54)	49–96	W = 235.50, p = .93, r = .02, 95% C.I [-8.00, 10.00]
Awareness		73.52 (11.85)	50–93		76.09 (14.53)	49–96	W = 193.00, p = .35, r = .14, 95% C.I [-12.00, 3.00]
Cognition		74.43 (11.08)	51–92		73.59 (14.88)	41–96	W = 229.50, p = .98, r = .01, 95% C.I -9.00, 9.00]
Communication		75.86 (13.60)	42–96		74.32 (12.85)	50–91	W = 245.00, p = .74, r = .05, 95% C.I -7.00, 10.00]
Motivation		66.29 (14.00)	44–95		65.59 (13.58)	41–89	W = 236.50, p = .90, r = .02, 95% C.I [-7.00, 10.00]
RRBs		80.57 (17.18)	48–108		78.59 (16.10)	48–106	W = 250.00, p = .65, r = .07, 95% C.I [-8.00, 14.00]

*Note.* 95% confidence intervals are for the difference in means/medians between the *DDX3X* and ID-comparison groups

^a^ The SIBs score was calculated by summing the raw scores of three DBC items related to self-injurious behaviour

*Autism characteristics.* Total T-scores on the SRS ranged from 46 to 98, with a mean score of 77.38 (SE = 14.08; a T-score of 75 indicates highly elevated levels of symptoms characteristic of autism). Four participants had scores that fell in the normal to mildly elevated range, four had scores in the moderately elevated range, and twelve had scores in the highly elevated range. Scores across subscales varied, with average levels of Social Awareness, Social Cognition, and Social Motivation falling within the mild-moderately elevated range, and average levels of Social Communication and Restricted, Repetitive Behaviours falling within the highly elevated range.

To explore autism characteristics of girls and women with *DDX3X* variants in more depth, we completed the ADI-R with the parents of seven participants. Based on published criteria, one participant met the current threshold for autism in all three domains (Socialisation, Language and Communication, Restricted and Repetitive Behaviour), while three met or exceeded cut-off scores on two of the three domains. Individual item scores on the ADI ranged from 0 to 3, with higher scores indicating greater evidence of autism characteristics. To assess whether there is consistency in specific autism characteristics within the *DDX3X* group, we reviewed average scores on each ADI item (Supplementary Information 3 – current behaviours, Supplementary Information 4 - scores at age 5, or the “most abnormal” time period). Items that had the highest endorsement (mean score > 1.5) related to difficulties with friendship and sharing (from the Social Development Domain), verbal intonation, reciprocal conversation, and imaginative play (from the Language and Communication Domain), and auditory sensitivity (from the Interests and Behaviours Domain). Conversely, items that were seldom endorsed (mean score < 0.5) related to making social overtures, offering comfort to others, and responding to other children (Social Development Domain), communicative speech, idiosyncratic use of language, and non-verbal gestures (Language and Communication Domain), and preoccupations, compulsions, and inflexibility (Interests and Behaviours Domain).

*Emotional and behavioural difficulties.* DBC scores varied widely in the *DDX3X* group, with Total Problem Behaviour T-scores (stratified by ID severity) ranging from 36 to 106. Thirteen of the twenty-one participants who completed the DBC had total scores that exceeded the clinical cut-off, indicating severe concerns across emotional and behavioural domains. Among the subscales, Anxiety was most commonly rated as an area of Serious concern (16/21 participants). Of note, the Anxiety subscale of the DBC captures behaviours relevant to both traditional clinical diagnoses (e.g., generalized or social anxiety disorder, specific phobias), as well as more distinct manifestations of anxiety among individuals with neurodevelopmental disorders (e.g., worries about routine). Anxiety-related behaviours that were frequently endorsed included shyness and social withdrawal, marked worries about routine, and unusual or excessive fears of sounds or objects (including fire alarms, babies crying, hand driers, sneezing, fireworks, onions, or butterflies - for one participant, fears of animals with stings had transformed into an obsession). The Self-Absorbed subscale, which largely captures autism-associated behaviours, was also commonly rated as an area of Serious concern (15/21 participants). Items frequently endorsed on this scale related to arranging objects in a strict order and sensory-seeking behaviours.

As noted above, thirteen participants displayed self-injurious behaviours (SIBs). During the MHI, parents reported that their daughters responded to stressful or frustrating situations by pulling their hair, biting their hands or knees, banging their head, or throwing themselves onto the floor. To further investigate the types and frequencies of SIBs demonstrated by participants, we also examined three items of the DBC pertaining to these behaviours: 11 participants banged their heads, 13 participants hit or bit themselves, and 13 participants picked or scratched their skin.

### Aim 2: Comparing adaptive, social and emotional characteristics in the *DDX3X* and ID-comparison groups

**Fig. 1:**
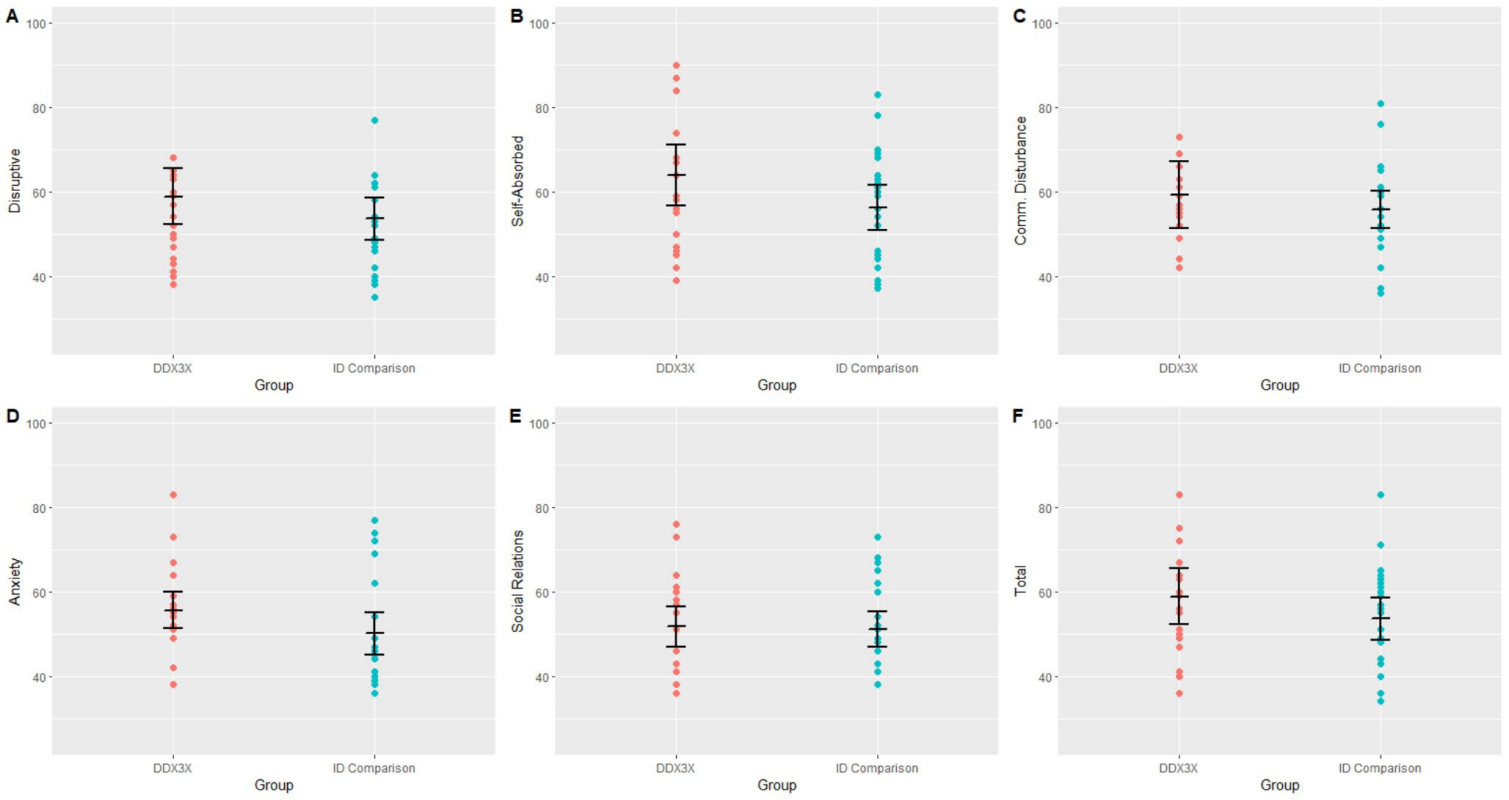
Emotional and behavioural difficulties across the *DDX3X* and ID-Comparison groups. Note: Individual data points represent DBC T-scores, stratified by ID severity. Error bars represent mean scores with 95% confidence intervals

Next, we explored whether any adaptive, social or emotional characteristics consistently differentiated the *DDX3X* group from girls and women with ID caused by variants in other genes, by comparing the *DDX3X* and ID-comparison groups using scores from the VABS, DBC, and SRS (Table 2). There were no significant differences between the two groups on the VABS or the SRS. On the DBC, the *DDX3X* group had higher scores on the Disruptive, Self-Absorbed, Communication, and Anxiety subscales, with effect sizes ranging from small to medium (Fig. 1). Furthermore, the group difference on the Anxiety subscale was significant (*W* = 333.50, *p* = .027).

To follow-up on parent reports of SIBs in the *DDX3X* group, we also explored whether rates of SIBs (as measured by three items on the DBC) were elevated in this group relative to the ID-comparison group. The *DDX3X* group demonstrated significantly higher levels of overall SIBs (*W* = 326.50, *p* = .04). In addition, a higher proportion of participants in the *DDX3X* group showed head banging (χ^2^ = 5.98, p = .014) and self-hitting/biting (χ^2^ = 4.39, *p* = .036). Skin picking/scratching was more common in the *DDX3X* group, but not significantly different from the comparison group (χ^2^ = 3.24, *p* = .072).

### Aim 3: Within-group predictors of social and emotional outcomes

Our third aim was to examine relationships between age, ID severity (VABS Composite), autism characteristics (SRS Total), anxiety (DBC Anxiety), and SIBs within the *DDX3X* and ID-Comparison groups separately. Bivariate correlations are displayed in Table 3 and plotted in Supplementary Information 5.

**Table 3:** Correlation matrix for the *DDX3X* and ID-Comparison groups

		Age		Adaptive Ability		Anxiety		Autism characteristics	
**DDX3X**	**ID-Comp.**								
**Adaptive Ability**		− 0.48*	− 0.40						
*Fisher’s test*		z = − 0.28, p = .39							
**Anxiety**		0.15	0.05	− 0.48*	− 0.04				
*Fisher’s test*		z = 0.31, p = .38		z = -1.46, p = .07					
**Autism characteristics**		− 0.17	− 0.40	− 0.42	− 0.23	0.68**	0.43*		
*Fisher’s test*		z = 0.74, p = .23		z = − 0.64, p = − .26		z = 1.09, p = .14			
**Self-Injury**		0.03	0.06	− 0.58**	− 0.15	0.62**	0.08	0.53*	0.09
*Fisher’s test*		z = − 0.09, p = .45		z = -1.60, p = .06		z = 2.00, p = .02		z = 1.49, p = .07	

* *p* < .05

** *p* < .01

In both groups, older age was associated with lower adaptive ability, and higher anxiety was associated with elevated autism characteristics. At the same time, Fisher’s tests revealed several significant between-group differences in correlation coefficients. Within the *DDX3X* group, higher level of anxiety was associated with lower adaptive ability, whilst self-injurious behaviour was associated with lower adaptive ability, higher anxiety and elevated autism characteristics. Regression analyses were carried out to examine interaction effects where Fisher’s tests highlighted possible group-specific associations. For the dependent variable anxiety, we found no significant interaction between group and adaptive function. For the dependent variable self-injury, we found significant or near significant interactions between group and adaptive function, anxiety and autism characteristics (Supplementary Information 6).

Finally, we ran three general linear models to explore whether genetic variant type influences commonly endorsed social and emotional features (autism characteristics, anxiety and SIBs) within the *DDX3X* group (Table 4). In all three models, we included age and adaptive ability as covariates, as participants with missense variants were on average older than those with protein-truncating variants (missense: mean age = 16.08 years, SE = 1.95; protein-truncating: mean age = 11.76 years, SE = 1.49), and, as has been reported previously (Tang et al., 2021), participants with missense variants had lower adaptive abilities than those with protein truncating variants (missense: mean VABS Composite = 43.71, SE = 4.79; protein truncating: mean VABS Composite = 58.77, SE = 4.04). Across all three models, variant type did not significantly predict levels of autism characteristics, anxiety, or SIBs.

**Table 4:** General linear models examining the effect of DDX3X genetic variant type on social and emotional characteristics

Variable	ANOVA	Adjusted R^2^	F	p	ηp2
**Model 1: Autism characteristics (n = 19)**	F (3, 15) = 3.74, p = .04	0.31			
Age			5.95	0.03	0.28
Adaptive Ability			9.99	0.01	0.40
Variant Type			0.31	0.59	0.02
**Model 2: Anxiety (n = 18)**	F (3, 14) = 1.96, p = .17	0.15			
Age			0.93	0.35	0.06
Adaptive ability			4.22	0.06	0.23
Variant type			0.14	0.72	0.01
**Model 2: SIBs (n = 18)**	F (3, 14) = 3.32, p = .05	0.29			
Age			0.91	0.36	0.06
Adaptive ability			7.90	0.01	0.36
Variant type			0.00	0.96	0.00

## Discussion

In this study, we build on previous characterisations of girls and women with *DDX3X* variants, in order to enhance prognostication, management and support for families and individuals with this diagnosis. We focus on social development and mental health, as these areas of need have been highlighted in published literature (Lennox et al., 2020; Tang et al., 2021), but not yet deeply explored. Our primary objective was to determine whether the neurodevelopmental characteristics of this highly variable disorder are as expected for girls and women with ID of any cause, or are in any respects specific to *DDX3X*. Below, we highlight novel observations not previously reported in the literature on *DDX3X*, discuss evidence for specificity of neurodevelopmental vulnerabilities, and consider potential explanations for the co-occurrence of specific characteristics within this group.

By conducting medical history interviews with a relatively large group of parents and carers, we identified several common areas of difficulty which have not been emphasised in previous reports. These included notably high frequency of early life feeding difficulties and sensory difficulties, which are important for understanding the experiences of parenting a young child with *DDX3X*, and are potentially relevant to emerging cognitive and social-emotional traits. Of particular note, a very high proportion of parents reported symptoms of self-injurious behaviours in their daughters, which have not been reported previously in association with this genetic diagnosis. Furthermore, many parents described their daughter as having a strong desire to make friends, yet lacking the skills to develop and sustain friendships, and tending towards heightened self-consciousness in social situations. Importantly, we observed these social and emotional challenges across the wide spectrum of ages and adaptive abilities in the *DDX3X* group. In addition to the diversity of challenges reported, parents described many of their daughters’ strengths and qualities, including caring and friendly personalities, humour and laughter, love of dancing and swimming, and willingness to persevere in the face of challenges.

Motivated by information elicited via these semi-structured interviews, we proceeded to systematically evaluate the social and emotional characteristics of the *DDX3X* group using a variety of questionnaire and interview methods. For several individuals, adaptive social functioning (as assessed by the VABS) was an area of strength relative to other domains, and on average, social functioning fell within the mild impairment range. Nonetheless, SRS scores indicated that many individuals with *DDX3X* variants exhibit autism characteristics, with the range of SRS scores aligning with previous reports (Lennox et al., 2020; Tang et al., 2021). This apparent discrepancy between adaptive social functioning and autism characteristics (i.e., social communication difficulties and restricted, repetitive behaviours) has been indicated by prior research (Klin et al., 2007), and highlights the multidimensional nature of social development and ability. Moreover, SRS scores (total and subscales) did not differ between the *DDX3X* and ID-comparison groups, suggesting that overall likelihood of autism characteristics as assessed by this tool is not a discriminating feature of *DDX3X*. SRS subscale scores and ADI-R responses highlighted diverse social vulnerabilities, cutting across traditional autism domains. Previous research in girls and women with fragile X syndrome (FXS) suggests a similar profile of social difficulties, characterized by preserved empathy alongside social communication challenges and repetitive behaviours (Miller et al., 2021; Marlborough et al., 2021). In the context of this literature, our findings suggest that the social vulnerabilities observed in association with *DDX3X* variants reflect autism characteristics shared amongst girls and women with monogenic ID; however, further work is required to confirm this.

In contrast, anxiety scores significantly differentiated the *DDX3X* group from the comparison group. Rates of reported anxiety, which was a concern for a substantial majority of participants in our study, are considerably higher than previously reported by Tang et al. (2021). One possible explanation for this discrepancy is that the Child Behaviour Checklist (Achenbach & Rescorla, 2001) used in previous studies may be less sensitive than the DBC to anxiety in young people diagnosed with autism and/or ID (Kerns et al., 2020). Another striking and previously unreported feature of the *DDX3X* group was the high incidence of self-injurious behaviours (SIBs), reported as a current or recent distressing problem for more than half of participants, and significantly more common amongst the *DDX3X* group than the comparison group. In summary, anxieties and SIBs can be anticipated for this group, signalling elevated need for clinical psychology support during childhood and adolescence.

These findings suggest that although the *DDX3X* gene has received a great deal of interest for its association with autism, it is equally important to recognise and address co-occurring anxiety in this group. Indeed, features of anxiety and autism often overlap (e.g., sensory sensitivities, intolerance of uncertainty, social avoidance), such that anxiety symptoms can often be misattributed to autism in clinical practice (see Postorino et al., 2017 for review). The presentation and needs of autistic girls and women with ID are often under-recognized and unmet, adding further complexity (Cummins et al., 2020; Dean et al., 2017). To address this, we evaluated the associations between autism characteristics and anxiety within the *DDX3X* group and comparison ID group, finding that the strength of this association did not significantly differ between groups. There are several possible interpretations of this association. First, it may reflect similarity in concepts and questionnaire items between measures of autism and anxiety. Alternatively, autism and anxiety may be causally related, although the nature and direction of these relationships need to be established via longitudinal analysis. Turning again to the FXS literature, anxiety among girls and women with FXS can in part be attributed to autism-associated social difficulties (Bartholomay et al., 2019). Third, anxiety and autism scores may be highly correlated because of convergent mechanisms at neurobiological or cognitive levels. One possibility is that structural and functional abnormalities in the amygdala and hippocampus, regions subserving emotion regulation which are also found to have decreased volumes in DDX3X and FXS mouse models (Boitnott et al., 2021; Suvrathan et al., 2010), represent a transdiagnostic risk mechanism underlying the co-occurrence of autism and anxiety in multiple monogenic disorders. Future research should examine these possibilities, as well as the specificity of these associations and underlying mechanisms in different genetic diagnosis groups.

We also found that within the *DDX3X* group (only), adaptive function, anxiety and autism characteristics predicted levels of SIBs. These findings may point to a complex causal pathway for SIBs in individuals with *DDX3X* variants, as has been demonstrated among autistic individuals without ID (Moseley et al., 2019). A combination of individual, social and environmental factors are likely to underlie the occurrence of SIBs in people with ID (Oliver et al., 2017), and interpreting underlying causes often requires reporters to make inferences about an individual’s internal state (Summers et al., 2017). Future research on the temporality of SIBs in individuals with *DDX3X* variants may clarify whether these behaviours are triggered by anxiety, or by other factors such as communication difficulties, pain or frustration (Adams & Oliver, 2011). At a mechanistic level, it is also important to situate SIBs in the context of broader cognitive and behavioural characteristics associated with *DDX3X* variants. For example, it is possible that shared neural factors (e.g., those associated with emotion regulation) underlie social interactions, anxiety, impulsivity and sensory over- or under-reactivity, which converge to increase risk for multi-domain difficulties including SIBs. Interestingly, among girls and women with FXS, those who engage in SIBs are also more likely to have a diagnosis of autism as well as anxiety and sensory processing issues (Symons et al., 2010). Further systematic investigation of the neural correlates, behavioural antecedents, and consequences of SIBs may clarify the aetiology and function of these behaviours in individuals with *DDX3X* variants, thus informing targeted prevention and response approaches (Arron et al., 2011).

Finally, we investigated the relevance of *DDX3X* variant type to anxiety and SIBs. In line with previous reports (Lennox et al., 2020; Tang et al., 2021), individuals with missense variants had poorer adaptive function compared to those with protein-truncating variants. General linear models revealed that variant type did not predict autism characteristics, anxiety or SIBs. It is possible that genetic variant type may moderate developmental and behavioural factors contributing to these outcomes, but this could not be observed due to our small sample size and limited measures. On the other hand, true lack of association between variant type and social-emotional features may reflect the complex and multifactorial pathways influencing these mental health outcomes, not predictable by genetic test result even in combination with other information. Future research should investigate the functional impact of specific variants – which may inform quantitative predictions of neurobiological impact – and also investigate non-genetic contributions to socioemotional outcomes.

## Strengths, limitations and future directions

Strengths of this study included the systematic post-diagnostic assessment of adaptive, social and emotional characteristics in individuals diagnosed with *DDX3X* variants from multiple centres, and comparison to an ID group matched in gender, age and global adaptive function; however, heterogeneity within the comparison group in terms of causative gene functions and behavioural characteristics is also a limitation. Our sample size meant that some analyses may have been underpowered to detect significant between-groups effects and interactions; however, this is one of the larger systematic studies of behavioural characteristics associated with this rare genetic diagnosis to date. Nonetheless, larger, multi-site studies should replicate and substantiate these findings. In addition, we relied primarily on parent report questionnaires and interviews, which can be limited in scope, sensitivity and specificity. Future studies incorporating multi-informant perspectives, clinician-administered interview and observational schedules, and cognitive and physiological assessments that are tailored for individuals with ID will contribute richer insights into cognitive, social and emotional functioning in individuals with *DDX3X* variants. Related to this, we did not examine how early and ongoing medical or physiological conditions (e.g., GI, sleep, motor issues) may impact developmental outcomes. This study included only females with *DDX3X* variants, and future research should determine whether a similar profile of social and emotional experiences is present in males. In addition, this was a cross-sectional study, and participants’ ages spanned a broad range. Longitudinal studies delineating changes in adaptive, socioemotional and neuropsychological functioning will be crucial for mapping the developmental progression of *DDX3X*-associated difficulties and identifying periods and mechanisms of risk and resilience.

## Conclusions

*DDX3X* variants are increasingly recognised as a common cause of neurodevelopmental disorders in females, with numbers of diagnosed individuals expected to rise as availability of testing increases. In this study, we expanded our knowledge of the social and emotional experiences of girls and young women with *DDX3X* variants, and highlighted a range of features that warrant clinical attention and further investigation. In common with other genomic disorders, it is important to place this diagnosis and its variable expression in the broader context of cognitive, psychological and interpersonal development. We find that levels of autism characteristics do not differentiate *DDX3X* and ID-comparison groups, but anxiety and self-injury levels are higher in the *DDX3X* group. Autism characteristics, anxiety and self-injury are closely linked within the *DDX3X* group and are not explained by global adaptive function. Future research investigating the diversity of mechanisms contributing to mental health vulnerabilities will guide the development of more precise information and support available to individuals with *DDX3X* variants and their families, and contribute to a better understanding of autism and mental health amongst girls and women with ID more broadly.

## Electronic Supplementary Material

Below is the link to the electronic supplementary material.

Supplementary Material 1

## Data Availability

The data that support the findings of this study are available to other ethically approved research projects from the corresponding author, KB at kate.baker@mrc-cbu.cam.ac.uk.

## Declarations

### Author Note

1. Elise Ng-Cordell – Department of Psychology, University of British Columbia, Vancouver, Canada; MRC Cognition and Brain Sciences Unit, University of Cambridge, United Kingdom. 2. Anna Kolesnik-Taylor – MRC Cognition and Brain Sciences Unit, University of Cambridge, United Kingdom. 3. Sinéad O’Brien – MRC Cognition and Brain Sciences Unit, University of Cambridge, United Kingdom. 4. Duncan Astle – MRC Cognition and Brain Sciences Unit, University of Cambridge, United Kingdom. 5. Gaia Scerif – Department of Experimental Psychology, University of Oxford, United Kingdom. 6. Kate Baker – MRC Cognition and Brain Sciences Unit, University of Cambridge, United Kingdom; Department of Medical Genetics, University of Cambridge, United Kingdom.

## List of abbreviations

*DDX3X*: DEAD-box helicase 3 X-linked.
*ID*: intellectual disability.
*ADHD*: Attention Deficit/Hyperactivity Disorder.
*MHI*: Medical History Interview.
*VABS*: Vineland Adaptive Behaviour Scales.
*SRS*: Social Responsiveness Scale, second edition.
*DBC*: Developmental Behaviour Checklist, second edition.
*ADI-R*: Autism Diagnostic Interview, Revised.
*SIB*: Self injurious behaviour.
*FXS*: Fragile X Syndrome.
