# Supplementary material for: Social and emotional characteristics of girls and young women with DDX3X-associated intellectual disability: a descriptive and comparative study
Source: J Autism Dev Disord. 2022 May 10;53(8):3208–19. doi: 10.1007/s10803-022-05527-w (PMC9087164; doi:10.1007/s10803-022-05527-w)
Supplement: Supplementary file 1 — Supplementary Material 1 [file 10803_2022_5527_MOESM1_ESM.docx]

**Social and emotional characteristics of girls and young women with DDX3X-associated intellectual disability: A descriptive and comparative study**

Elise Ng-Cordell^1,2^, Anna Kolesnik-Taylor^2^, Sinéad O’Brien^2^, Duncan Astle^2^, Gaia Scerif^3^, Kate Baker^2,4^

1. Department of Psychology, University of British Columbia, Vancouver

2. MRC Cognition and Brain Sciences Unit, University of Cambridge

3. Department of Experimental Psychology, University of Oxford

4. Department of Medical Genetics, University of Cambridge

Corresponding author: Kate Baker, [kate.baker@mrc-cbu.cam.ac.uk](mailto:kate.baker@mrc-cbu.cam.ac.uk)

**Supplementary Material**

Supplementary Information 1. Genetic diagnoses in the ID-comparison group

Supplementary Information 2. Participants’ gene variant information

Supplementary Information 3. ADI score profiles – Current

Supplementary Information 4. ADI score profiles – Most Abnormal / age 5

Supplementary Information 5. Scatterplots for within-group correlations

Supplementary Information 6. Regression models with group and interaction terms

**Supplementary Information 1. Genetic diagnoses in the ID-comparison group**

| **Gene** | **N** |
| --- | --- |
| *ARID1B* | 3 |
| *CASK* | 1 |
| *CTNNB1* | 1 |
| *DYRK1A* | 1 |
| *EHMT1* | 3 |
| *GRIN2A* | 1 |
| *KAT6B* | 1 |
| *SETD5* | 4 |
| *SHANK1* | 2 |
| *STXBP1* | 5 |
| *TRIO* | 1 |
| **Total** | 23 |

**Supplementary Information 2. Participants’ gene variant information**

| **Participant no.** | **Decipher/100K Genome ID** | **Inheritance** | **Variant type** | **Amino acid change** | **Nucleotide change** | **Pathogenicity** | **ID severity** |
| --- | --- | --- | --- | --- | --- | --- | --- |
| 1 | 272869 | De novo | Missense | p.Cys467Tyr | c.1400G>A | Likely pathogenic | Severe |
| 2 | 273466 | De novo | Splice (acceptor) | - | c.46-2A​>G | Likely pathogenic | Mild |
| 3 | 272597 | De novo | Stop-gained | p.Ser24Ter | c.71C​>A | Pathogenic | Mild |
| 4 | 264193 | De novo | Stop-gained | p.Arg291Ter | c.871C​>T | Likely pathogenic | Mild |
| 5 | 261890 | Inherited | Missense | p.Arg533His | c.1598G​>A | Likely pathogenic | Moderate |
| 6 | 267102 | De novo | Missense | p.Ala392Pro | c.1174G​>C | Likely pathogenic | Severe |
| 7 | 267105 | De novo | Missense | p.Ala392Pro | c.1174G​>C | Likely pathogenic | Moderate |
| 8 | 261162 | De novo | Stop-gained | p.Arg45Ter | c.133C​>T | Pathogenic | Moderate |
| 9 | 290561 | De novo | Splice (donor) | - | c.281+1G​>C | Likely pathogenic | Moderate |
| 10 | 300283 | De novo | Missense | p.Arg474Cys | c.1420C​>T | Likely pathogenic | Mild |
| 11 | - | - | - | - | - | - | Moderate |
| 12 | 277307 | De novo | Splice (donor) | - | c.45+1G>T | Pathogenic | Mild |
| 13 | 303778 | De novo | Frameshift | p.Lys208SerfsTer13 | c.623delA | Pathogenic | Borderline |
| 14 | - | De novo | Missense | Cys341Arg | c.1021T>C | - | Severe |
| 15 | 282969 | De novo | Splice donor | - | c.45+1G>A | Pathogenic | Profound |
| 16 | - | - | - | - | - | - | Moderate |
| 17 | - | De novo | Splice (acceptor) |  | c.766-8_766-2del | Likely pathogenic | Mild |
| 18 | - | - | - | - | - | - | Moderate |
| 19 | - | Unknown | Frameshift | p.Asn124LysfsTer5 | c.372_373delCA | - | Mild |
| 20 | 307552 | De novo | Frameshift | p.Phe83Ter | c.241_242insCTAG | Pathogenic | Borderline |
| 21 | 210015169 | De novo | Frameshift | p.Gly153Leufs*69 | c.455_456dupCT | Pathogenic | Mild |
| 22 | 281511 | De novo | Missense | p.Ser542Leu | c.1625C>T | Likely pathogenic | Moderate |
| 23 | - | Unknown | Splice site | - | c.765+1G>A | Pathogenic | Mild |

**Supplementary Information 3. ADI score profiles - Current**

**
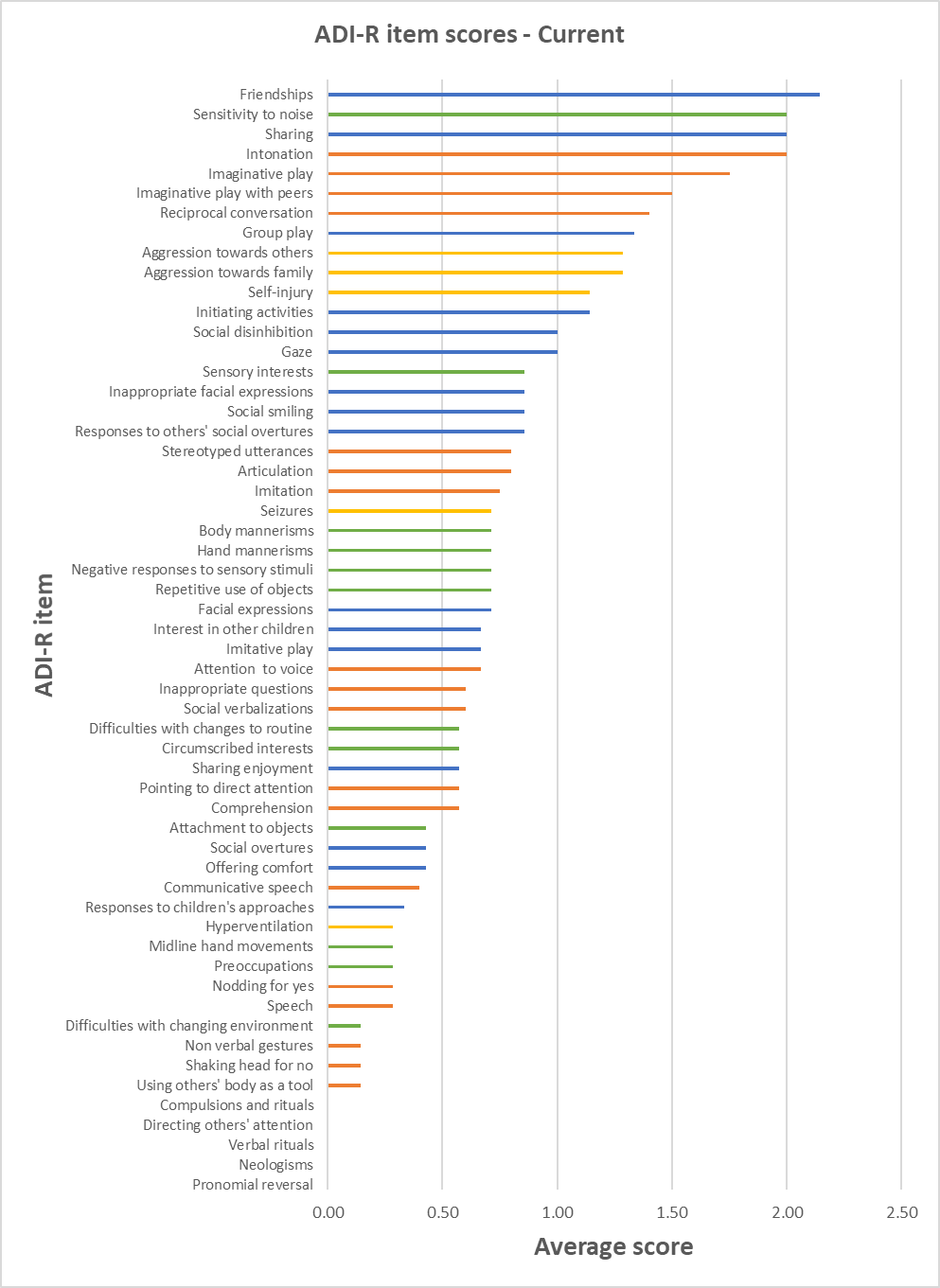
**

*Note.* Orange items = Language and Communication, Blue = Social Development and Play, Green = Interests and Behaviours, Yellow = General Behaviours.

**Supplementary Information 4. ADI score profiles – Most Abnormal / age 5**


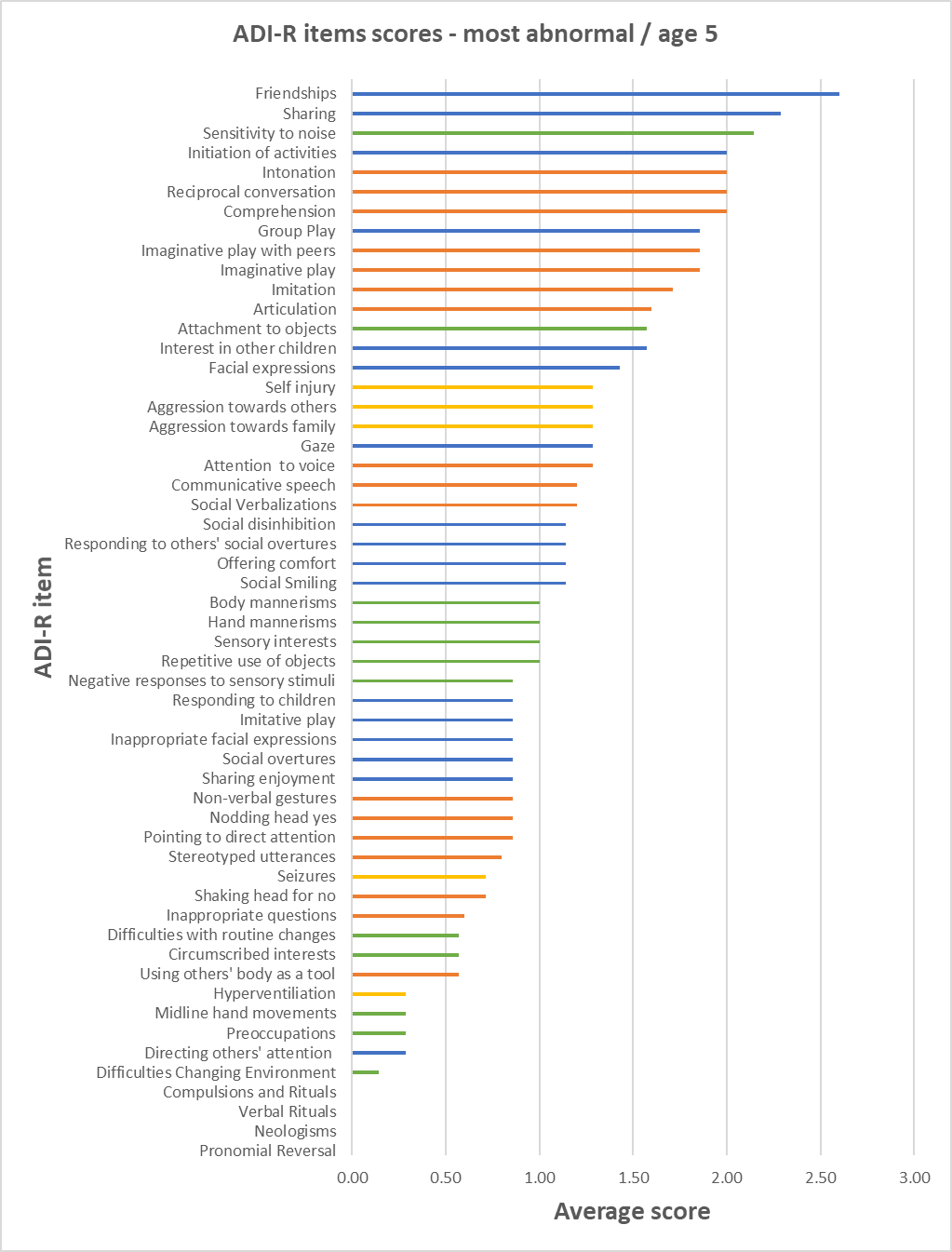


*Note.* Orange items = Language and Communication, Blue = Social Development and Play, Green = Interests and Behaviours, Yellow = General Behaviours.

**Supplementary Information 5. Scatterplots for within-group correlations**


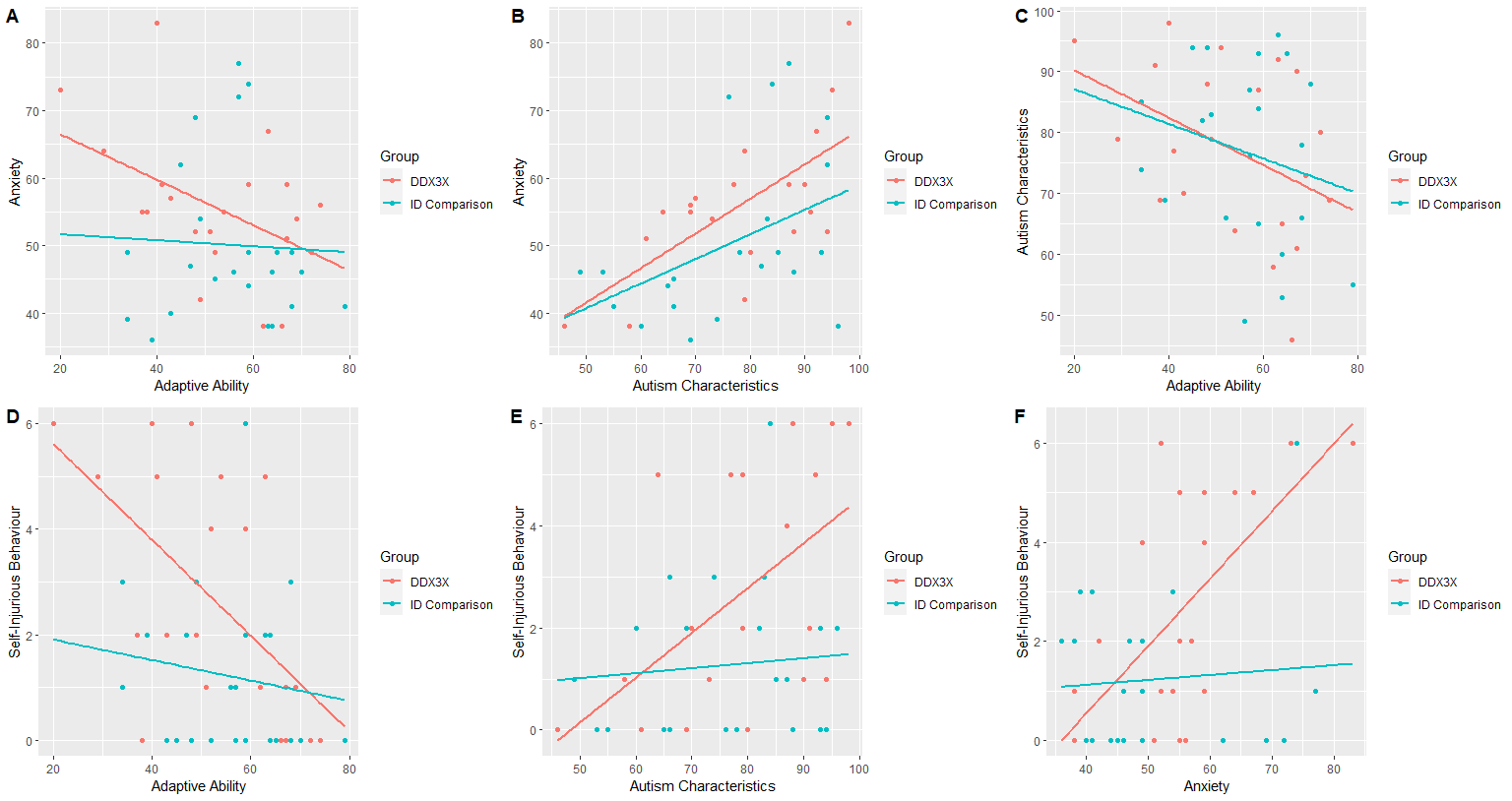


**Supplementary Information 6. Regression Models with Group and Interaction Terms**

| **Dependent variable**  Predictors | **ANOVA** | **Adjusted R^2^** | **B** | **Std. Err** | **Beta (β)** | **t** | **p** | **95% CI for B** |
| --- | --- | --- | --- | --- | --- | --- | --- | --- |
| **Anxiety (n = 44)** | F (3,40) = 2.25, p =.10 | .14 |  |  |  |  |  |  |
| Group |  |  | -20.96 | 14.71 | -.90 | -1.41 | .17 | [-5.45, 9.02] |
| Adaptive ability |  |  | -.63 | .39 | -.72 | -1.60 | .12 | [-1.42, .17] |
| Group* Adaptive ability |  |  | .29 | .26 | .89 | 1.11 | .27 | [-.24, .83] |
| **Self-Injury (n = 44)** | F (3,40) = 6.68, p =.001 | .28 |  |  |  |  |  |  |
| Group |  |  | -5.13 | 2.28 | -1.26 | -2.25 | .03 | [-9.74, -.51] |
| Adaptive ability |  |  | -.16 | .06 | -1.05 | -2.66 | .01 | [-.29, -.04] |
| Group*Adaptive ability |  |  | .07 | .04 | 1.24 | 1.74 | .09 | [-.01, .15] |
| **Self-Injury (n = 44)** | F (3,40) = 7.39, p <.001 |  |  |  |  |  |  |  |
| Group |  |  | 5.62 | 2.55 | 1.38 | 2.20 | .03 | [.46, 10.78] |
| Anxiety |  |  | .26 | .08 | 1.49 | 3.35 | .002 | [.10, .42] |
| Group*Anxiety |  |  | -.13 | .05 | -1.81 | -2.69 | .01 | [-.22, -.03] |
| **Self-Injury (n = 42)** | F (3,38) = 4.85, p =.006 |  |  |  |  |  |  |  |
| Group |  |  | 4.76 | 3.16 | 1.17 | 1.51 | .14 | [-1.64, 11.16] |
| Autism char. |  |  | .17 | .07 | 1.14 | 2.55 | .02 | [.03, .30] |
| Autism char.*Self Injury |  |  | -.08 | .04 | -1.69 | -1.94 | .06 | [-.16, .00] |
| *Note*. Method = Enter. | | | | | | | | |
